# rs744166 Polymorphism of the *STAT3* Gene Is Associated with Risk of Gastric Cancer in a Chinese Population

**DOI:** 10.1155/2014/527918

**Published:** 2014-04-23

**Authors:** Kexin Yuan, Huimin Liu, Lina Huang, Xiyun Ren, Jingjing Liu, Xiaoqun Dong, Wenjing Tian, Yunhe Jia

**Affiliations:** ^1^Department of Epidemiology, College of Public Health, Harbin Medical University, 197 Xuefu Road, Harbin, Heilongjiang 150081, China; ^2^Department of Neurology, The Forth Affiliated Hospital, Harbin Medical University, Harbin, Heilongjiang 150001, China; ^3^Department of Biomedical and Pharmaceutical Sciences, College of Pharmacy, The University of Rhode Island, Pharmacy Building, 7 Greenhouse Road, Kingston, RI 02881, USA; ^4^Department of Colorectal Cancer Surgery, The Third Affiliated Hospital, Harbin Medical University, 149 Xuefu Road, Harbin, Heilongjiang 150081, China

## Abstract

The aim of this study was to explore the association between polymorphisms in signal transducer and activator of transcription protein 3 (*STAT3*) and the risk of gastric cancer. In the present study, a case-control study was conducted in which rs2293152 and rs744166 polymorphisms in *STAT3* were analyzed in 209 Chinese patients with gastric cancer and 294 cancer-free controls. The genotypes were determined by polymerase chain reaction restriction fragment length polymorphism method. For the rs744166 polymorphism, the TC genotype (adjusted OR = 0.60, 95% CI = 0.39–0.92, and *P* = 0.020) and CC genotype (adjusted OR = 0.41, 95% CI = 0.21–0.80, and *P* = 0.009) were associated with a decreased risk of gastric cancer compared to the TT genotype. However, rs2293152 did not show any difference in gastric cancer risk between patients and controls in the CG/CC genotype compared to the GG genotype. Besides, the SNP effects were additive to the effects of environmental factors without any interaction between them in the susceptibility to gastric cancer. Collectively, rs744166 polymorphism might be significantly associated with a decreased risk of gastric cancer in a Chinese population. Additionally, polymorphisms in *STAT3*, along with environmental factors, might be associated with the development of gastric cancer.

## 1. Introduction

In recent years, gastric cancer (GC) incidence rates have decreased substantially in most parts of the world. However, GC was still the fourth most common malignancy worldwide, with approximately 989,600 new cases, particularly in Eastern Asian countries, such as China [1, 2]. It is well known that the development of GC is a multifactorial process that includes both host polymorphisms and environmental factors [3].

Signal transducer and activator of transcription 3 (STAT3) is a key transcription factor of the Janus kinase (JAK)/signal transducer and activator of transcription (STAT) signaling pathway [4, 5]. The JAK/STAT pathway transmits a wide range of regulatory factors that modulate gene transcription, including cytokines, growth factors, and hormones [6]. In particular, STAT3 can be activated by a variety of ligands that respond to massive signals such as IL-6, TNF-*α*, and VEGF [4, 5, 7, 8]. Aberrant expression and constitutive activation of STAT3 are involved in a broad range of human malignancies, including gastric, breast, prostate, and nonsmall cell lung cancers [9–12]. Recent studies have identified STAT3 activation as a key event in regulating cell growth, motility, migration, invasion, angiogenesis, and immune response in GC [8, 9, 13–17]. Furthermore, it was reported that* Helicobacter pylori* (*H. pylori*) CagA protein activated the STAT3 signaling pathway in gastric cells, providing a potential mechanism by which chronic* H. pylori* infection promotes the development of GC [18, 19].

*STAT3* is located on chromosomal region 17q21. To date, single-nucleotide polymorphisms (SNPs) in* STAT3* have shown significant associations with cervical cancer, nonsmall cell lung cancer, leukemia, prostate cancer, and hepatocellular cancer [20–24]. Ferguson et al. also found that* STAT3* SNPs are significantly associated with susceptibility of Crohn's disease [25]. To our knowledge, no studies have been published to explore the association between* STAT3 *polymorphisms and GC risk until now.

In this case-control study we aim to evaluate the associations between* STAT3* polymorphisms and susceptibility of GC, as well as discussing potential environmental factors.

## 2. Materials and Methods

### 2.1. Study Subjects

A case-control study was conducted using a Chinese study population of 209 GC patients and 294 controls. All patients, based on pathologic diagnosis, were recruited from the Third Affiliated Hospital of Harbin Medical University. For the frequency-matched controls on age and sex, 154 healthy individuals were recruited from Harbin Center for Disease Control, as well as 140 cancer-free patients who were chosen from the neurology department at the Fourth Affiliated Hospital of Harbin Medical University as controls. All cases and controls had completed a face-to-face questionnaire. A 5 mL sample of venous blood was collected from each subject following the interview. Cases and controls with incomplete questionnaires, as well as non-GC patients, were excluded from this study. Informed consent was obtained from all subjects and the protocol was approved by the Human Research and Ethics Committee of Harbin Medical University.

### 2.2. Data Collection

Demographic and habit related data such as age, gender, smoking (including cigarette and pipe) and drinking history, family history of cancer, and frequency of food consumption were collected using a structured questionnaire. We defined smokers as those who smoked more than one cigarette/pipe per day for at least half a year. Likewise, drinkers were defined as those who consumed two or more alcoholic drinks per week for at least half a year. For family history, it referred to first and second degree relatives (parents, grandparents, siblings, and offspring).

### 2.3. SNP Selection and Genotyping

Genomic DNA was extracted from whole blood using a QIAamp DNA Blood Mini Kit (Qiagen, Germany). DNA purity and concentrations were determined by spectrophotometric measurement of absorbance at 260/280 nm.

*STAT3* gene polymorphisms were reviewed based on previously published literature which identified functional effects or associations with disease. Minor allele frequency (MAF) of ≥5% in the Asian population, parameters set by the SNP database of the National Center for Biotechnology Information, were also reviewed. Ultimately, rs2293152 (G>C) and rs744166 (T>C) polymorphisms were selected.

Polymerase chain reaction restriction fragment length polymorphism (PCR-RFLP) was implemented to determine the genotypes. The PCR primers were designed using Primer Premier 5.0 software. For rs2293152, the primer sequences were 5′-TAGAGGCTTCCTTTTGTTCCG-3′ (forward) and 5′-CCAGTTGTCTTTCATCCC-3′ (reverse) that generated the 356-bp fragment. For rs744166, the primer sequences were 5′-GAGTACAAACCCTGAACC-3′ (forward) and 5′-GACTTGGTGACTGACTGAA-3′ (reverse) that generated the 301-bp fragment. Amplification was performed under the following conditions: an initial denaturation for 5 min at 95°C, followed by 30 cycles of denaturation at 95°C for 30 s, annealing at 56°C for 30 s, and extension at 72°C for 30 s, followed by a final extension at 72°C for 7 min. The amplified fragments of* STAT3* rs2293152 and rs744166 were digested by restriction enzymes AciI and AluI (New England BioLabs), respectively, 5 units for 16 h at 37°C, followed by electrophoresis on a 2% agarose gel.

We used the common genotype homozygotes, the rare genotype homozygotes, and the heterozygous of the polymorphisms (rs2293152 and rs744166) for direct sequencing.

### 2.4. Statistical Analysis

A chi-square (*χ* ^2^) test was used to evaluate Hardy-Weinberg equilibrium (HWE), as well as comparing the genotype and allele frequencies between groups. The associations between genetic polymorphisms and the risk of GC were estimated by computing the odds ratios (ORs) and their 95% confidence intervals (CIs) from unconditional univariate and multivariate logistic regression analyses. We evaluated the interactions between genetic polymorphisms and environmental factors on the risk of GC with four types of ORs (OR_e_, OR_g_, OR_eg_, and OR_i_). Reference groups consisted of individuals who were not exposed to environmental factors and lacked genetic variants. OR_e_ compares the risk of GC based on the level of exposure to environmental factors; OR_g_ is the odds ratio for the association between the genetic variant with the risk of GC; OR_eg_ is the odds ratio for the combined effect of both genetic variants and environmental factors; OR_i_ is the odds ratio of interactions of genetic variants and environmental factors. (OR_i_ = OR_eg_/OR_e_OR_g_, OR_i_ > 1 indicated a positive interaction (synergy); OR_i_ < 1 indicated a negative interaction (antagonism); OR_i_ = 1 indicated that there was no interaction.) A *P* value of <0.05 was considered statistically significant. All tests were two-sided, and all statistical analyses were conducted with SAS 9.1 (SAS Institute, Cary, North Carolina, USA).

## 3. Results

### 3.1. Characteristics of Study Subjects

This case-control study enrolled 209 GC patients and 294 cancer-free controls. The demographic information on study subjects are shown in Table 1. There were no significant differences between cases and controls in the distributions of age and sex (*P* > 0.05). However, smoking and drinking were overrepresented in the cases compared to the controls (*P* < 0.001), and cases with family history of cancer were more frequent than controls (*P* < 0.001).

### 3.2. Association between STAT3 Polymorphisms and GC

In our study population, the alleles at rs2293152 and rs744166 loci were in HWE (*P* = 0.233 for rs2293152 and *P* = 0.851 for rs744166). The genotype distributions of these two SNP loci in the cases and controls are presented in Table 2. The distributions of the genotypes for* STAT3* rs744166 were 9.1% (TT), 42.6% (TC), and 48.3% (CC) in the cases and 36.4% (TT), 48.3% (TC), and 15.3% (CC) in control subjects (*P* = 0.01). Additionally,* STAT3* rs744166 showed a statistically significant association in further logistic regression analysis. Compared to the TT genotype carriers, the TC genotype (adjusted OR = 0.60, 95% CI = 0.39–0.92, and *P* = 0.020) and CC genotype (adjusted OR = 0.41, 95% CI = 0.21–0.80, and *P* = 0.009) carriers were both less frequent among cases than controls, suggesting a dominant effect of the C allele. Overall, C carriers (either TC or CC) were associated with a lower GC risk when compared to those carrying the TT genotype (adjusted OR = 0.55, 95% CI = 0.37–0.83, and *P* = 0.004). Conversely, the genotype and allele frequencies of locus rs2293152 did not show a significant difference between GC cases and controls.

### 3.3. Stratified Analysis of STAT3 Polymorphism rs744166 and GC Risk

We further evaluated the association between genotypes of these two selected SNPs of* STAT3* and GC risk by stratified subgroups of age, sex, smoking status, drinking consumption, and family history of cancer, assuming a dominant genetic model (Table 3). Stratified analysis revealed that a decreased risk of GC was associated with the rs744166 TC+CC genotypes among subjects 60 years of age and older (adjusted OR = 0.49, 95% CI = 0.26–0.90, and *P* = 0.021), but not in subjects less than 60 years old. Additionally, TC+CC genotypes were associated with a decreased risk of GC in male subjects (adjusted OR = 0.54, 95% CI = 0.32–0.88, and *P* = 0.014), whereas the association was not statistically significant in female subjects. When stratified by smoking status, the C carriers were found to be significantly associated with GC (adjusted OR = 0.54, 95% CI = 0.32–0.93, and *P* = 0.026) compared to the TT genotype carriers in smokers. Likewise, drinkers with C carriers showed a significantly decreased risk of GC (adjusted OR = 0.38, 95% CI = 0.21–0.70, and *P* = 0.002), compared to those carrying the TT genotype. Stratification by family history of cancer revealed a significant association of TC+CC genotypes and the risk of GC (adjusted OR = 0.55, 95% CI = 0.36–0.85, and *P* = 0.007) in subjects without family history of cancer, whereas the association was not statistically significant in subjects with family history of cancer. Conversely, we observed no statistical significance between the rs2293152 gene polymorphism and the risk of GC (data not show).

### 3.4. Combined Effects of STAT3 Polymorphisms on GC Risk

Table 4 shows analyses of interaction and combination effects between genotypes and environmental exposures for the risk of GC. We found that these two SNPs effects were additive to the effects of environmental factors without any interaction between them in the susceptibility to GC.

For rs744166, significant combination effects between high intake of chicken (≥average 2 times/week) and fresh fruit (≥average 2 times/week) and the TC+CC genotypes were observed (chicken: OR_eg_ = 0.25 and 95% CI = 0.13–0.48; fresh fruit: OR_eg_ = 0.34 and 95% CI = 0.19–0.61). Consumption of seafood (≥average 2 times/week) and using a refrigerator to store food were associated with a decreased risk of GC in subjects with a C allele or TT genotype. On the contrary, long-term irregular eating habits were associated with an increased risk of GC. Additionally, no statistically significant interactions or combination effects were observed between consumption of pork, preference for hot food, and the rs744166 polymorphism on GC risk. Moreover, the combination effects of rs2293152 and environmental factors are almost identical to rs744166 (data shown in Table 4).

## 4. Discussion

GC is an inflammation-related disease that induces massive cytokine release, including IL-1, IL-6, IL-12, and TNF-*α* [26–28]. STAT3, which transmits a wide range of cytokines, was first identified in 1994 as an IL-6-activated acute-phase response factor (APRF) [29]. It is frequently reported to be overexpressed in various cancers [30] and has, therefore, been recognized as an oncogene. Several* STAT3* SNPs were reported to be significantly associated with cervical cancer, nonsmall cell lung cancer, metastatic renal cell carcinoma, prostate cancer, and hepatocellular cancer. These include rs2293152, rs744166, rs4769793, rs4796793, and rs7211777 [4, 20, 23, 24]. Moreover, rs744166 was also reported to have a significant association with Crohn's disease [25, 31]. Based on previous studies, our case-control study detected the effect of* STAT3* rs2293152 and rs744166 gene polymorphisms on GC. In our study, we found that subjects with the minor C allele of rs744166 had a decreased risk of developing GC compared to the common genotype in a Chinese population. However, we did not find any significant association between the rs2293152 gene polymorphism and risk of GC.

Recently, increasing evidence shows that polymorphisms of the* STAT3* gene are associated with various diseases [21, 31–33]. In a large German cohort study, a significant association was observed between the minor allele of* STAT3* rs744166 and Crohn's disease (OR = 0.83, 95% CI = 0.688–0.998, and *P* = 0.04) [31]. Similarly, Danoy et al. identified* STAT3* rs744166 as a susceptible loci of ankylosing spondylitis (OR = 0.84, 95% CI = 0.77–0.91, and *P* = 2.6 × 10^−5^) in a population of white European ancestry from the UK, USA, Canada, and Australia [34]. Moreover, Jiang et al. reported that the minor allele of rs744166 significantly decreased the risk of nonsmall cell lung cancer in a Chinese population [20]. These findings on the rs744166 C allele and the decreased risk of GC in the present study are consistent with reported results. However, the results of our study on rs2293152 show variation from previous research. A Chinese study involving 1,021 hepatocellular carcinoma patients and 1,012 healthy controls found that rs2293152 (GG versus CC) was significantly associated with risk of hepatocellular carcinoma [24]. Yet, other researches on familial breast cancer and nonsmall cell lung cancer did not show associations of rs2293152 [20, 33] and neither did our study. These differences could potentially be the result of varying environmental backgrounds, ethnic groups, and sample sizes.

Furthermore, our present study revealed a decreased effect with the C allele of rs744166 in subgroups of males, smokers, and drinkers. Stratified analysis suggested that the association between the C allele of rs744166 and the risk of GC was more profound in males than females. A previous study reported that noncardia cancer was more common in males than females by a ratio of approximately 2 : 1, and gastric cardia cancer had a ratio of nearly 4.1 : 1 in a Chinese population [35]. Our data suggests that* STAT3* polymorphism may play an important role in males with GC. Smoking, which generates reactive oxygen species production and induces DNA adducts that may lead to mutations and thus the initiation of carcinogenesis, has been confirmed to be an independent risk factor of GC [36, 37]. Meanwhile, for drinking, previous reports suggested that alcohol consumption is one of the risk factors of GC as well [38, 39]. In our present study, it is also suggested that the influence of the C allele of rs744166 on GC was more critical in subgroups of smokers and drinkers. The underlying mechanism involved in the association between* STAT3* and smoking and drinking is not clear. It is likely that smoking and drinking might significantly induce* STAT3* expression, and it is possible that the C allele of rs744166 leads to a higher basal expression level of* STAT3* under unnormal circumstances. Therefore, the subjects carrying the C allele of rs744166 might not have decreased GC risk under normal conditions but might have a reduced risk when exposed to tobacco and alcohol that might induce* STAT3* expression in response.

Interestingly, we found that the influence of C allele of rs744166 disappeared in the subjects with family history, although it existed in all study subjects. Positive family history was reported as a strong and consistent risk factor for GC due to the combination result of a number of alleles [40, 41]. Therefore, the decreased risk effect of rs744166 in subjects with family history may be overwhelmed by the role of familial aggregation, which may partly contribute to the results we observed. Small sample size in the subgroups may also account for the variation seen between studies. Therefore, further studies should be conducted with a larger sample size.

Single SNPs in low-penetrance genes are unlikely to significantly affect susceptibility to cancer, but combination effects may exist [42, 43]. In our study, significant combination effects were observed with environmental factors. Fruits are high in antioxidants, phytosterols, and other substances that may inhibit carcinogenesis by free-radical quenching or by blocking the formation of N-nitroso compounds. Epidemiologic studies have found that fruits are associated with a decreased risk of GC [44]. In this study, we found a significant combination effect between fresh fruit consumption and the C allele of rs744166 in decreasing the risk of GC. Meanwhile, lifestyle choices such as long-term irregular eating habits and the methods of storing food may also be important factors for GC in this Chinese population. To our knowledge, this is the first study to be concerned with the interaction between the* STAT3* gene polymorphisms and environmental factors for the risk of GC; further studies are needed to confirm this phenomena and a mechanistic explanation for our findings needs to be explored.

Some limitations in our study need to be addressed. First, the relatively small sample size may reduce the statistical power in stratified analysis. The second limitation was an inevitable recall bias during data collection in regard to environmental factors, although measures were taken to minimize this bias. Third, we adopted a frequency questionnaire to collect information about dietary intake that did not take into consideration the amounts of food being consumed. Therefore, the questionnaire had limited power to detect precise gene-dietary interactions. Finally,* H. pylori* is an independent risk factor for GC that we did not explore due to ethical considerations. These related issues will need to be addressed in our future studies.

In conclusion, our data provided the first evidence that the C allele of* STAT3 *rs744166 is associated with decreased GC risk in a Chinese population. The minor C allele of rs744166 may correlate with* STAT3* expression and thus exert a protective effect on GC development. Additional large-scale, well-designed studies that include environmental factors are required to further validate the role of* STAT3* gene polymorphisms in GC risk.

## Figures and Tables

**Table 1:** Baseline characteristics of study subjects.

Characteristics	Cases (*N* = 209)	Controls (*N* = 294)	*P* value*
	No. (%)	No. (%)	
Age (years)			0.331
<60	106 (50.7)	162 (55.1)	
≥60	103 (49.3)	132 (44.9)	
Sex			0.076
Male	158 (75.6)	201 (68.4)	
Female	51 (24.4)	93 (31.6)	
BMI^a^ (kg/m^2^)			
Mean ± SD	22.4 ± 3.8	24.2 ± 3.3	<0.001
Smoking status^a^			<0.001
Never	71 (34.1)	154 (52.6)	
Ever	137 (65.9)	139 (47.4)	
Alcohol consumption			<0.001
Nondrinkers	85 (40.7)	172 (58.5)	
Drinkers	124 (59.3)	122 (41.5)	
Family history of cancer^ab^			<0.001
No	157 (77.0)	280 (95.9)	
Yes	47 (23.0)	12 (4.1)	
TNM stage^a^			
I	14 (7.3)		
II	15 (7.8)		
III	142 (74.0)		
IV	21 (10.9)		

BMI: body mass index (weight/height^2^).

*P value from two-sample *t* tests and *χ* ^2^ tests.

^a^Missing data: BMI: 7; smoking: 2; family history of cancer: 7; tumor site: 17.

^b^Family history of cancer: gastric, esophageal, liver, colorectal, and other cancers.

**Table 2:** Genotype and allele frequencies of STAT3 polymorphisms and their associations with GC risk.

Genotypes	Cases (*N* = 209)	Controls (*N* = 294)	OR (95% CI)	*P* value	OR_adj_ (95% CI)^a^	*P* value
	Number (%)	Number (%)				
rs2293152						
GG	56 (26.8)	74 (25.2)	1.00		1.00	
CG	94 (45.0)	157 (53.4)	0.79 (0.51–1.22)	0.287	0.87 (0.54–1.41)	0.540
CC	59 (28.2)	63 (21.4)	1.24 (0.75–2.03)	0.400	1.40 (0.81–2.42)	0.330
CG/CC			0.92 (0.61–1.38)	0.681	1.03 (0.66–1.62)	0.893
rs744166						
TT	101 (48.3)	107 (36.4)	1.00		1.00	
TC	89 (42.6)	142 (48.3)	0.66 (0.45–0.97)	0.035	0.60 (0.39–0.92)	0.020
CC	19 (9.1)	45 (15.3)	0.45 (0.25–0.82)	0.009	0.41 (0.21–0.80)	0.009
TC/CC			0.61 (0.43–0.88)	0.008	0.55 (0.37–0.83)	0.004

OR: odds ratio; CI: confidence interval.

^a^Adjusted for age, sex, smoking, drinking, BMI, and family history of cancer.

**Table 3:** Stratified analyses of rs744166 genotypes between GC cases and controls.

Variables	rs744166		*P* value
	TT	TC+CC	
Age (years)			
<60			
Cases/controls	48/61	58/101	
OR_adj_ (95% CI)^a^	1.00	0.64 (0.37–1.13)	0.127
≥60			
Cases/controls	53/46	50/86	
OR_adj_ (95% CI)^a^	1.00	0.49 (0.26–0.90)	0.021
Sex			
Male			
Cases/controls	77/73	81/128	
OR_adj_ (95% CI)^a^	1.00	0.54 (0.32–0.88)	0.014
Female			
Cases/controls	24/34	27/59	
OR_adj_ (95% CI)^a^	1.00	0.63 (0.28–1.40)	0.254
Smoking status^b^			
Never			
Cases/controls	32/58	39/96	
OR_adj_ (95% CI)^a^	1.00	0.56 (0.29–1.07)	0.081
Ever			
Cases/controls	68/48	69/91	
OR_adj_ (95% CI)^a^	1.00	0.54 (0.32–0.93)	0.026
Alcohol consumption			
Nondrinkers			
Cases/controls	39/69	46/103	
OR_adj_ (95% CI)^a^	1.00	0.73 (0.40–1.31)	0.288
Drinkers			
Cases/controls	62/38	62/84	
OR_adj_ (95% CI)^a^	1.00	0.38 (0.21–0.70)	0.002
Family history of cancer^bc^			
No			
Cases/controls	76/102	81/178	
OR_adj_ (95% CI)^a^	1.00	0.55 (0.36–0.85)	0.007
Yes			
Cases/controls	22/4	25/8	
OR_adj_ (95% CI)^a^	1.00	0.50 (0.11–2.28)	0.371

OR: odds ratio; CI: confidence interval.

^a^Adjusted for age, sex, smoking, drinking, BMI, and family history of cancer.

^b^Missing data: smoking: 2; family history of cancer: 7.

^c^Family history of cancer: gastric, esophageal, liver, colorectal, and other cancers.

**Table 4:** Interactive effects of *STAT3* polymorphisms and environmental exposures on GC risk.

Environmental exposures	rs2293152		Interaction		rs744166		Interaction	
	GG	CG+CC			TT	TC+CC		
	OR_eg_ (95% CI)^a^		OR_i_ (95% CI)^a^	*P* value	OR_eg_ (95% CI)^a^		OR_i_ (95% CI)^a^	*P* value
Pork								
≤3 times/week	1.00	0.99 (0.54–1.84)	0.92 (0.36–2.32)	0.85	1.00	0.65 (0.37–1.15)	0.72 (0.31–1.65)	0.44
>3 times/week	1.60 (0.72–3.59)	1.46 (0.79–2.69)			1.78 (0.95–3.36)	0.83 (0.46–1.51)		
Chicken								
<2 times/week	1.00	1.03 (0.54–1.96)	1.16 (0.45–2.97)	0.75	1.00	0.71 (0.38–1.32)	0.55 (0.24–1.28)	0.17
≥2 times/week	0.43 (0.19–0.97)	0.52 (0.28–0.96)			0.65 (0.34–1.22)	0.25 (0.13–0.48)		
Seafood								
<2 times/week	1.00	1.00 (0.58–1.75)	1.20 (0.40–3.57)	0.75	1.00	0.61 (0.37–1.01)	0.65 (0.24–1.72)	0.38
≥2 times/week	0.21 (0.08–0.55)	0.25 (0.13–0.50)			0.31 (0.15–0.63)	0.12 (0.06–0.25)		
Fresh fruits								
<2 times/week	1.00	0.91 (0.49–1.72)	1.21 (0.47–3.08)	0.70	1.00	0.60 (0.34–1.04)	0.89 (0.39–2.06)	0.79
≥2 times/week	0.51 (0.22–1.14)	0.56 (0.29–1.07)			0.65 (0.34–1.22)	0.34 (0.19–0.61)		
Long-term irregular eating habit								
No	1.00	1.07 (0.63–1.82)	0.64 (0.17–2.43)	0.52	1.00	0.54 (0.34–0.87)	1.49 (0.47–4.75)	0.50
Yes	8.18 (2.56–26.1)	5.63 (2.63–12.1)			4.59 (1.92–11.0)	3.72 (1.69–8.19)		
Preference for hot food								
No	1.00	0.96 (0.52–1.75)	1.09 (0.43–2.79)	0.86	1.00	0.58 (0.34–0.99)	0.95 (0.41–2.24)	0.91
Yes	1.74 (0.78–3.89)	1.82 (0.96–3.47)			1.90 (0.99–3.62)	1.04 (0.58–1.88)		
Using refrigerator to store food								
No	1.00	0.71 (0.34–1.49)	1.98 (0.73–5.35)	0.18	1.00	0.76 (0.39–1.46)	0.55 (0.23–1.31)	0.18
Yes	0.15 (0.06–0.37)	0.22 (0.11–0.44)			0.36 (0.18–0.69)	0.15 (0.08–0.28)		

OR: odds ratio; CI: confidence interval.

^a^Adjusted for age, sex, smoking, drinking, BMI, and family history of cancer.
